# Elucidating the molecular physiology of lantibiotic NAI-107 production in *Microbispora* ATCC-PTA-5024

**DOI:** 10.1186/s12864-016-2369-z

**Published:** 2016-01-12

**Authors:** Giuseppe Gallo, Giovanni Renzone, Emilia Palazzotto, Paolo Monciardini, Simona Arena, Teresa Faddetta, Anna Giardina, Rosa Alduina, Tilmann Weber, Fabio Sangiorgi, Alessandro Russo, Giovanni Spinelli, Margherita Sosio, Andrea Scaloni, Anna Maria Puglia

**Affiliations:** Laboratory of Molecular Microbiology and Biotechnology, STEBICEF Department, University of Palermo, 90128 Palermo, Italy; Proteomic and Mass Spectrometry Laboratory, ISPAAM, National Research Council, 80147 Naples, Italy; Naicons srl, 20139 Milan, Italy; The Novo Nordisk Foundation Center for Biosustainability, Technical University of Denmark, 2970 Hørsholm, Denmark; German Center for Infection Research (DZIF) partner site Tübingen, 72074 Tübingen, Germany; Sistema Informativo di Ateneo (SIA), Area Servizi di Rete, University of Palermo, 90128 Palermo, Italy

**Keywords:** Actinomycetes, Antibiotic production, Differential proteomics, 2D-DIGE and mass spectrometry, Metabolic pathways, Regulatory network, Molecular and cellular functions

## Abstract

**Background:**

The filamentous actinomycete *Microbispora* ATCC-PTA-5024 produces the lantibiotic NAI-107, which is an antibiotic peptide effective against multidrug-resistant Gram-positive bacteria. In actinomycetes, antibiotic production is often associated with a physiological differentiation program controlled by a complex regulatory and metabolic network that may be elucidated by the integration of genomic, proteomic and bioinformatic tools. Accordingly, an extensive evaluation of the proteomic changes associated with NAI-107 production was performed on *Microbispora* ATCC-PTA-5024 by combining two-dimensional difference in gel electrophoresis, mass spectrometry and gene ontology approaches.

**Results:**

*Microbispora* ATCC-PTA-5024 cultivations in a complex medium were characterized by stages of biomass accumulation (A) followed by biomass yield decline (D). NAI-107 production started at 90 h (A stage), reached a maximum at 140 h (D stage) and decreased thereafter. To reveal patterns of differentially represented proteins associated with NAI-107 production onset and maintenance, differential proteomic analyses were carried-out on biomass samples collected: i) before (66 h) and during (90 h) NAI-107 production at A stage; ii) during three time-points (117, 140, and 162 h) at D stage characterized by different profiles of NAI-107 yield accumulation (117 and 140 h) and decrement (162 h). Regulatory, metabolic and unknown-function proteins, were identified and functionally clustered, revealing that nutritional signals, regulatory cascades and primary metabolism shift-down trigger the accumulation of protein components involved in nitrogen and phosphate metabolism, cell wall biosynthesis/maturation, lipid metabolism, osmotic stress response, multi-drug resistance, and NAI-107 transport. The stimulating role on physiological differentiation of a TetR-like regulator, originally identified in this study, was confirmed by the construction of an over-expressing strain. Finally, the possible role of cellular response to membrane stability alterations and of multi-drug resistance ABC transporters as additional self-resistance mechanisms toward the lantibiotic was confirmed by proteomic and confocal microscopy experiments on a *Microbispora* ATCC-PTA-5024 lantibiotic-null producer strain which was exposed to an externally-added amount of NAI-107 during growth.

**Conclusion:**

This study provides a net contribution to the elucidation of the regulatory, metabolic and molecular patterns controlling physiological differentiation in *Microbispora* ATCC-PTA-5024, supporting the relevance of proteomics in revealing protein players of antibiotic biosynthesis in actinomycetes.

**Electronic supplementary material:**

The online version of this article (doi:10.1186/s12864-016-2369-z) contains supplementary material, which is available to authorized users.

## Background

The filamentous actinobacterium *Microbispora* ATCC-PTA-5024 is a potentially relevant industrial bacterial strain since it produces the lantibiotic NAI-107 [1, 2], an antibiotic peptide active against Gram-positive bacteria - including methicillin-resistant *Staphylococcus aureus* (MRSA), glycopeptide-intermediate *S. aureus* (GISA) and vancomycin-resistant enterococci (VRE) - and some Gram-negative bacteria [3, 4]. In *Microbispora* ATCC-PTA-5024, the *mlb* cluster has been demonstrated to contain genes encoding enzymes required for NAI-107 biosynthesis, pathway specific regulators, and proteins involved in secretion and self-resistance mechanism (also referred as cell immunity) [5, 6]. The NAI-107 cluster is highly similar to the *mib* cluster present in *M. corallina* NRRL 30420, where the function of the corresponding gene products and the mechanisms regulating gene expression have been elucidated [7, 8]. Similarly to other lantibiotics [9], NAI-107 is ribosomally synthesized as a precursor peptide that is encoded by the *mlbA* structural gene. The NAI-107 precursor undergoes extensive modifications, including the formation of *meso*-lanthionine (Lan) or 3-methyllanthionine (Me-Lan) residues, before the removal of the N-terminal leader peptide and the secretion of the final 24 amino acid-long product.

In *Streptomyces coelicolor*, the most studied actinomycete used as model strain, the biosynthesis of antibiotics and other secondary metabolites is elicited as a developmental program and a physiological response to a variety of environmental stimuli and conditions, which depend on cell density, nature and/or quantity of carbon, nitrogen and phosphate sources [10–14]. From a molecular cell biology perspective, production of these secondary metabolites is generally associated with a cascade of regulatory proteins, which span from those playing pleiotropic roles to pathway-specific regulators controlling anabolic or catabolic genes and, eventually, gene products responsible for antibiotic biosynthesis. A comprehensive understanding of such a complex network of signalling, regulatory circuits and biochemical reactions may be in principle difficult, labour intensive and time consuming. Nowadays, the continuous integration of bioinformatic tools and holistic technologies has allowed the development of consolidated strategies to manage the huge amounts of molecular information on gene expression and biochemical capabilities deriving from “omic” investigations, ultimately leading to novel perspectives and approaches to explore microbial strain physiology [15]. In this context, proteomics has recently been used for shedding light on the relationships among overall metabolic pathways and biosynthesis of interesting bioactive molecules in several actinomycete strains [16–23].

In the perspective of using *Microbispora* strains for the industrial synthesis of antibiotics effective against multidrug-resistant Gram-positive pathogens, insights on the molecular cell physiology of this rare genus of filamentous actinobacteria would be beneficial to develop robust and economically-feasible production processes. Thus, due to the promising applicative scenario of NAI-107 and the limited knowledge on *Microbispora* ATCC-PTA-5024, an extensive investigation on the proteomic changes associated with lantibiotic production was carried out on the wild-type (WT) strain at different fermentation stages by using combined two-dimensional difference *in gel* electrophoresis (2D-DIGE) and mass spectrometry (MS) approaches. In addition, with the aim to confirm results concerning the impact of NAI-107 on *Microbispora* physiology, comparative proteomic experiments were performed on a *Microbispora* ATCC-PTA-5024 lantibiotic-null producer strain after the exposure to NAI-107. All proteomic data were integrated with bioinformatic, confocal microscopy and genetic engineering results in order to elucidate regulatory networks, biochemical pathways and molecular processes linking bacterial growth and NAI-107 production, thus providing the first global functional picture of a member of the *Microbispora* genus.

## Results

### Growth parameters and NAI-107 production kinetics

**Fig. 1:**
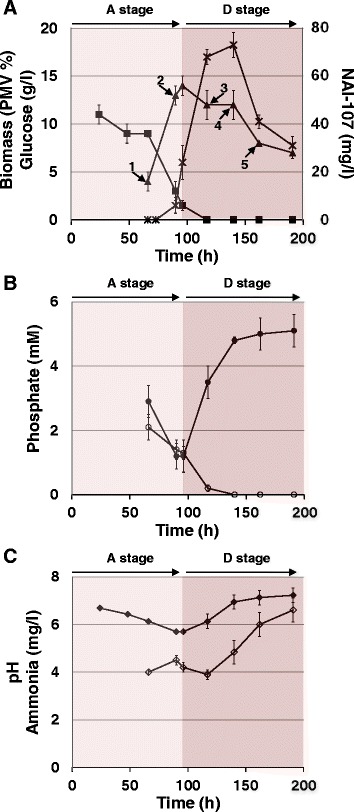
Growth parameters of *Microbispora* ATCC-PTA-5024 WT strain. Each value is the mean of three independent measurements performed on parallel cultivations. Error bars represent standard deviation. Kinetic profiles during cultivation time of: glucose concentration, bacterial biomass and NAI-107 production yields (panel **a**); inorganic (Pi) and organic (Po) phosphate concentration (panel **b**); pH values and ammonia concentration (panel **c**). Squares: glucose Triangles: percentage of packed mycelium volume (PMV %). Asterisks: NAI-107. Black circles: Pi. Empty circles: Po. Black diamonds: pH. Empty diamonds: ammonia. In A panel, numbered arrows represent time points of biomass sampling: 1, A-66 h; 2, A-90 h; 3, D-117 h; 4, D-140 h; 5, D-162 h

When incubated in the KV6 complex medium, *Microbispora* ATCC-PTA-5024 WT showed a growth kinetics characterized by a stage of active mycelial cell growth and biomass accumulation (A), ranging from A-66 h to A-96 h, which was followed by a stage of general cell growth arrest and cellular lysis occurrences, as inferred by biomass content decline (D) from D-117 h to D-191 h (Fig. 1a). The A and D stages of biomass accumulation profiles paralleled the kinetics of medium concentration changes of glucose, phosphate and ammonium as well as of pH variation during cultivation time (Fig. 1). In particular, glucose resulted progressively consumed and then completely exhausted at D-117 h (Fig. 1a). The medium inorganic phosphate (Pi) content gradually decreased until A-90 h, reaching the lowest value, and then progressively increased during D substages (Fig. 1b). The content of hydrolysable phosphate from medium organic sources (Po) showed a decreasing trend during the A substages that became more rapid during the transition from A to D substages where Po resulted almost exhausted at D-117 h and completely exhausted thereafter. The medium ammonia content resulted slightly fluctuating until D-117 h and then gradually increased (Fig. 1c). In agreement with glucose consumption and the ammonia concentration profile, the medium pH was characterized by a bi-phasic profile (Fig. 1c) comprising: i) a first stage of acidification ending at A-96 h, which is probably a consequence of the production of organic acids from glycolysis and tricarboxylic acid (TCA) cycle [24, 25]; ii) a second stage of pH increase, which is probably due to ammonia produced by medium peptide hydrolysis and amino acid utilization as carbon and nitrogen source during growth [24–26]. NAI-107 production was observed starting from A-90 h, when the glucose concentration in the medium was about 3 g/l (Fig. 1a). NAI-107 accumulation still occurred at D-117 and D-140 h, when NAI-107 production reached the maximum yield. At D-162 h and thereafter, NAI-107 amount dropped down (Fig. 1a).

### Experimental design of the proteome analyses of *Microbispora* ATCC-PTA-5024 WT strain

Differential proteomic analyses were accomplished by comparing whole protein extracts from WT strain biomass samples collected: i) at A-66 and A-90 h time-points, corresponding to A substages preceding and following the lantibiotic production onset, respectively, (Fig. 1a); ii) at D-117, D-140 and D162 h time-points, when NAI-107 production yield firstly reaches a maximum and then progressively decreases (Fig. 1a). Thus, Additional file 1: Table S1 reports the quantitative proteomic changes between A-66 and A-90 h substages, detailing the molecular events associated with or leading to NAI-107 production in a context characterized by an active biomass accumulation supported by glucose utilization. On the other end, Additional file 2: Table S2 illustrates the quantitative variations between D-117, D-140 and D-162 h substages, showing the cellular protein complement associated with the maintenance, first, and the decline, then, of NAI-107 production in a context of glucose exhaustion and biomass content decrement. In addition, since it has been previously demonstrated that the genetic regulatory circuits controlling the biosynthesis of the lantibiotic nisin [27] and nisin biosynthesis itself [28] partly occur at the membrane level, a proteomic comparison between A-66 and A-90 h substages was also carried out on ad hoc prepared bacterial membrane extracts. Additional file 3: Table S3 shows the corresponding results for protein quantification and identification.

The time sampling of the A and D stage differential proteome analyses was corroborated by Principal Component Analysis (PCA) performed on protein abundance 2D-patterns and by the overall diminished representation of primary metabolism enzymes during D substages, as detailed in the “Additional file 4: Supplementary Results section and in Additional file 5: Figure S1 and S2. The reliability of quantitative proteomic results was demonstrated by the coherent trend of the amount of protein species encoded by genes organized as putative operons, as detailed in the “Additional file 4: Supplementary Results section. *Microbispora* ATCC-PTA-5024 proteomic 2D-maps, representative of the above-mentioned experimental conditions, are provided in a digitalized database built as an interactive webpage available at http://www.unipa.it/ampuglia/microbispora/. A detailed description of this database is provided in the “Additional file 4: Supplementary Results section.

### Comparative proteomic analysis between A substages preceding and following the lantibiotic production onset

**Fig. 2:**
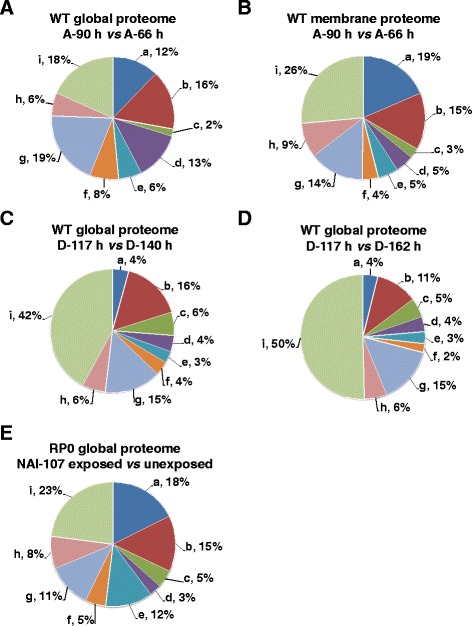
Functional distribution of the differentially represented protein species at the A and D stages. Functional distribution of the differentially represented protein species in the whole extract from the proteome comparison A-90 h *vs* A-66 h (panel **a**). Functional distribution of the differentially represented protein species in the membrane extract from the proteome comparison A-90 h *vs* A-66 h (panel **b**). Functional distribution of the differentially represented protein species in the whole extract from the proteome comparison D-117 h *vs* D-140 h (panel **c**). Functional distribution of the differentially represented protein species in the whole extract from the proteome comparison D-117 h *vs* D-162 h (panel **d**). Functional distribution of the differentially represented protein species in the whole extract from RP0 cells exposed to NAI-107, with respect to unexposed cells used as control (panel **e**). a: amino acid metabolism; b: carbon metabolism; c: energy metabolism; d: nucleotide metabolism; e: others; f: oxidoreduction and oxidative stress; g: protein metabolism; h: secondary metabolism; i: unknown function

**Fig. 3:**
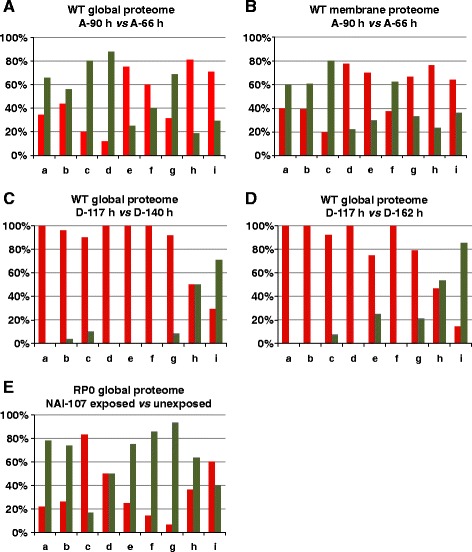
Quantitative trend of the differentially represented protein species at the A and D stages according to their distribution in protein functional classes. Percentage depiction of over- (red bars) and down- (green bars) represented protein species: i) in the whole extracts at A-90 h with respect to A-66 h (panel **a**); ii) in the membrane extracts at A-90 h with respect to A-66 h (panel **b**); iii) in the whole extracts at D-117 h with respect to D-140 h (panel **c**); iv) in the whole extracts at D-117 h with respect to D-162 h (panel **d**); v) in the whole extract in the RP0 cells exposed to NAI-107 with respect to unexposed cells used as control (panel **e**). a: amino acid metabolism; b: carbon metabolism; c: energy metabolism; d: nucleotide metabolism; e: others; f: oxidoreduction and oxidative stress; g: protein metabolism; h: secondary metabolism; i: unknown function

**Fig. 4:**
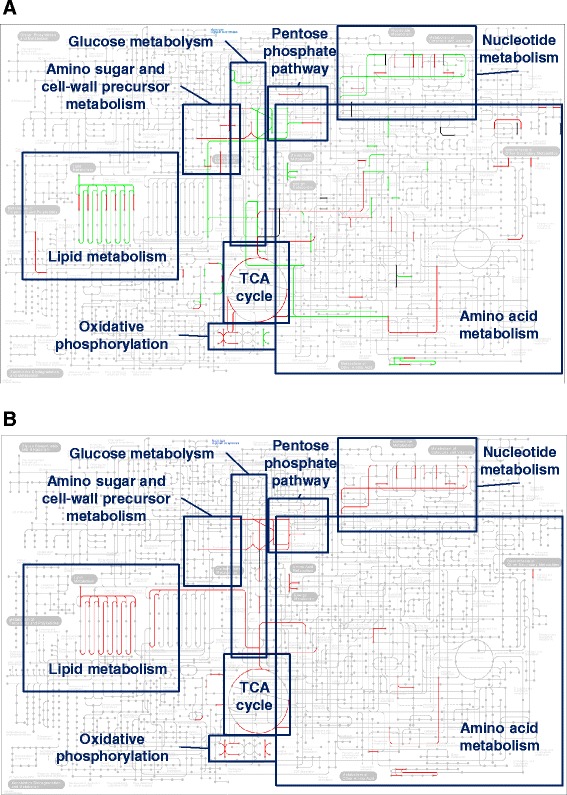
Global view of the metabolic pathways at the A and D stages. Metabolic pathways involving proteins over- and under-represented at A-90 h (panel **a**) or at D-117 h (panel **b**) are shown with red and green colour, respectively. Interactive maps can be obtained by following instructions present at the *Microbispora* ATCC-PTA-5024 proteome web page (http://www.unipa.it/ampuglia/microbispora/). The matabolic maps were obtained by using KEGG Pathway mapping tool [29]

Proteomic analysis of the whole protein extracts revealed 271 differentially represented protein species. Among that, 125 increased and 146 decreased their abundance in the biomass sampled at A-90 h, with respect to the one taken at A-66 h (Additional file 1: Table S1). On the other hand, proteomic analysis of the membrane protein extracts revealed 196 differentially represented spots; among that, 110 increased and 86 decreased their abundance in the above-mentioned comparison (Additional file 3: Table S3). Protein spots from both experiments were analyzed by MS procedures, which assigned them to specific ORFs in the *Microbispora* ATCC-PTA-5024 genome [5]. Corresponding proteins were then clustered into nine groups (a-i; Fig. 2a and b) according to the predicted functions, based on BLAST interrogations against the KEGG2 database [29]. Spots corresponding to multiple protein identification (16 in total) (Additional file 6: Table S5) were excluded from functional analysis. Gene products associated with secondary metabolism were grouped also considering the information deriving from a previous bioinformatic analysis [5, 30]. Both proteomic datasets, having 38 ORF products in common, showed proteins with unknown function (i) as the most represented group, followed by those involved in: i) amino acid, carbon, nucleotide and protein metabolism (a, b, d and g, respectively) in the case of global proteome analysis; ii) amino acid, carbon and protein metabolism (a, b and g, respectively) in the case of membrane proteome analysis. The distribution into functional groups of increased or decreased protein species from both proteomic datasets revealed similar profiles, with the exception of that involved in nucleotide and protein metabolism, oxidoreduction and oxidative stress response (f), which showed different quantitative trends at global and membrane levels (Fig. 3a and b). Thus, both membrane and global proteomic analyses revealed that functional groups including protein species involved in amino acid, carbon and energy metabolism were mostly down-regulated at A-90 h (Fig. 4a), while those containing components associated with secondary metabolism or having unknown function were mostly up-regulated at this time point. Some of the most interesting proteins presenting a differential abundance are detailed below within sub-paragraphs highlighting specific metabolic pathways and molecular processes, which are put in relationship with NAI-107 production onset.

#### Carbon metabolism

Most of the differentially represented enzymes involved in carbon metabolism showed a decreased abundance at A-90 h (Fig. 4a). In particular, as detailed in the “Additional file 4: Supplemetary Results section, almost all glycolytic enzymes and enzymes using glycolysis products as substrates showed a decreased abundance at A-90 h, in coincidence with the dropping down of the glucose concentration in the medium (Fig. 1a), which may be also related with a differential expression of genes involved in glycogen metabolism. In this context, the down-representation of two glycogen phosphorylase (GlgP) protein species, coupled with the over-representation of a corresponding proteolytic fragment, and the concomitant slight increment of the alpha amylase GlgE abundance at A-90 h suggested an increased glycogen conversion to trehalose, whose intracellular synthesis is associated with morpho-physiological development and stress resistance capability in streptomycetes [31, 32].

**Fig. 5:**
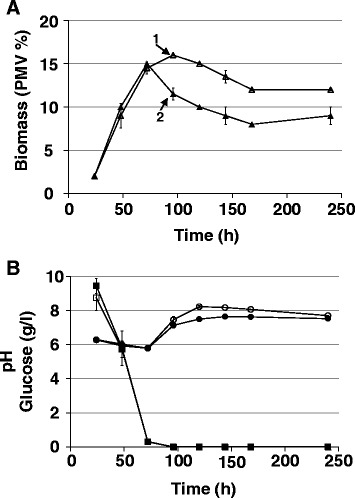
Growth kinetics parameters of the *Microbispora* ATCC-PTA-5024 RP0 strain. Biomass accumulation profiles of NAI-107 exposed (black triangles) and unexposed (empty triangles) cultivations measured as percentage of packed mycelium volume (PMV%) (panel **a**). NAI-107 was added at 72 h; pH and glucose concentration profiles of NAI-107 exposed (black circles and squares, respectively) and unexposed (empty circles and squares, respectively) cultivations (panel **b**). Numbered arrows in A panel represent biomass sampling: 1, unexposed cultivations; 2, cultivations 24 h exposed to NAI-107

Among the A-90 h over-represented proteins belonging to the carbon metabolism group, worth mentioning are some TCA cycle enzymes, such as citrate synthase, malate dehydrogenase and fumarate reductase iron-sulfur subunit (Additional file 1: Table S1 and Additional file 3: Table S3; Fig. 5a). Their up-regulation may be put in relation with an increased production of the alpha-keto acids deriving from the utilization of peptides and amino acids present in the medium. This possibility is supported by the over-representation at A-90 h of different protein species, which were further identified as two putative peptidases (NCBI accession numbers: ETK34477.1 and ETK32069.1) and four proteins with unknown function. An intensive-mode Phyre2 analysis [33] also designed the latter proteins as possible alkaline serine proteases (NCBI accession numbers: ETK32012.1 and ETK32013.1), peptidase (NCBI accession number: ETK32069.1) and trypsin-like protein (NCBI accession number: ETK31048.1), with a confidence greater than 90 % for a percentage identity spanning from 56 % to 69 %. Coherently with this context, ammonia concentration slightly increased moving from A-66 h to A-90 h substages (Fig. 1c) as a consequence of medium peptide hydrolysis and amino acid utilization [24–26].

Some enzymes involved in lipid metabolism were observed as differentially accumulated during A substages. Among that, worth mentioning is the down-representation of 3-oxoacyl-ACP synthase II (FabF) that is involved *de novo* lipid synthesis. FabF showed a coherent profile in both membrane and whole proteome analyses, which also paralleled the over-representation of proteins involved in: i) recycling of fatty acids, such as AB-hydrolase associated lipase that presented subtle quantitative changes in membrane proteome analysis); ii) lipid modifications, like the short-chain dehydrogenase/reductase SDR (FabG) and cyclopropane-fatty-acyl-phospholipid synthase (CFA).

#### Amino acid and amino sugar metabolism

Concerning proteins involved in amino acid metabolism, few enzymes resulted over-represented in coincidence of NAI-107 production at A-90 h. Among that, worth mentioning is glutamine synthetase (GlnA) (Additional file 1: Table S1), a key enzyme of nitrogen metabolism that catalyzes the biosynthesis of glutamine starting from glutamate and ammonia [34], whose concentration increased from A-66 to A-90 h. In agreement with this result, glutamine-fructose-6-phosphate transaminase (GlmS) was also over-represented at A-90 h (Additional file 1: Table S1); this enzyme catalyzes the first step of amino sugar biosynthesis, which leads to the synthesis of glucosamine-6P using glutamine as ammonia donor. Interestingly, increased levels of GFPT paralleled with the over-representation at A-90 h of diaminopimelate epimerase, UDP-N-acetylmuramate-alanine ligase and UDP-N-acetylmuramoylalanyl-D-glutamyl-2,6- diaminopimelate--D-alanyl-D-alanyl ligase, whose encoding genes are organized in putative operons (Additional file 4: Supplementary Results); these proteins are all involved in the biosynthesis of the cell wall stem peptide, thus revealing an up-regulation of this cellular process during NAI-107 production at A-90 h.

#### Protein metabolism

According to the general down-representation of enzymes involved in amino acid metabolism during NAI-107 production at A-90 h, protein components involved in protein biosynthesis, folding and export were generally under-represented in both global and membrane proteomic maps. On the other hand, many proteins associated with protein degradation and turnover resulted over-represented therein. A detailed description of those changes is provided in the Additional file 4: Supplementary Results section. The putative functional role of ETK34477.1, ETK32069.1, ETK32012.1, ETK32013.1, ETK32069.1, and ETK31048.1 proteases has been mentioned above.

#### Nucleotide metabolism

At A-90 h, proteomic analysis of whole bacterial extracts suggested a decreased representation of proteins involved in nucleotide metabolism, DNA replication and, in accordance with the observed down-regulation of protein biosynthesis, RNA synthesis. Interestingly, some enzymes involved in nucleotide metabolism were observed as associated with membranes too. The detailed description of the abundance profile of these proteins is provided in Additional file 4: Supplementary Results section; it is consistent with a global metabolic context of decreased nucleotide synthesis.

#### Proteins involved in nitrogen, phosphate and sulphur metabolism

The accumulation profile of key factors involved in phosphate, nitrogen and sulphur metabolism were positively associated with NAI-107 production and, in the first two cases, with the measured concentration profiles of Pi, Po (Fig. 1b) and ammonia (Fig. 1c) in the spent medium. This was evident from the augmented abundance levels detected at A-90 h for: i) PstS and PhoH proteins that are part of the Pho regulon, whose members are devoted to the scavenging for phosphate from organic sources and assimilation thereof [35, 36]; ii) the putatively co-transcribed gene products P-II (GlnK) and protein-PII uridylyltransferase (GlnD) (Additional file 4: Supplementary Results), and the above-mentioned GlnA, which ultimately control the cell utilization of ammonia [34]; iii) thiosulfate sulfurtransferase that is involved in sulphur metabolism.

#### Regulatory proteins of morpho-physiological differentiation

Some *bld*-like proteins [37] were detected as differentially represented in coincidence of NAI-107 production at A-90 h. In particular, the *Microbispora* lipoprotein BldK, which is an ABC-type permease involved in mycelium differentiation by permitting the transport of the morphogenic oligopeptide Bld261 in *S. coelicolor* [38], was found as under-represented in its two intact isoforms and over-represented in its putative proteolytic fragment to highlight a decreased need of its function during NAI-107 production. Instead, the anti-anti sigma factor BldG showed augmented levels in concomitance of NAI-107 biosynthesis, which well paralleled the moderate increment of the transcriptional regulator BldD. This result may be consistent with a synchronized progression of physiological regulatory events in *Microbispora* ATCC-PTA-5024, suggesting an early accumulation of BldK at A-66 h, whose abundance dropped down with the concomitant accumulation of BldG and BldD at A-90 h, according to the regulatory cascade proposed in *S. coelicolor* [37]. In *S. coelicolor*, the *bld* cascade has already been associated with a growth rate decrement due to nutrient limitation that activates the biosynthesis of the intracellular signalling molecule ppGpp(p) [13, 39]. In agreement with this observation, *Microbispora* ObgE GTPase showed a modest, but appreciable, abundance increment, while PNPase presented decreased abundance levels of intact species and augmented representation of the corresponding degradation products during NAI-107 production. The first protein is an important modulator of bacterial physiological differentiation by controlling the decrease of GTP pool, while the second one is an enzyme involved in mRNA degradation. In *S. coelicolor*, the ObgE homologue has been shown to control biomass production positively and bacterial differentiation negatively throughout ppGpp level regulation [40]. On the other hand, PNPase is negatively controlled by ppGpp in actinomycetes [41]. By describing the dynamics of the corresponding isoforms during NAI-107 production, this proteomic study suggests the occurrence of post-translational regulatory mechanisms determining decreased PNPase levels in concomitance of the antibiotic biosynthesis.

#### Secondary metabolism proteins

An accumulation of *mlb* gene products [5, 6] was observed at A-90 h. In particular, MlbF and MlbZ ATPases, which are specifically devoted to NAI-107 transport [7] and immunity [6] as members of two distinct ABC transporters, were found as membrane-associated proteins over-represented at A-90 h. A similar quantitative trend, although with subtle abundance changes, was observed for lantibiotic cyclase MlbC, which is involved in lanthionine ring formation [7]. Indeed, MlbF and MlbZ were also found in the global proteome analysis dataset at A-90 h (Additional file 1: Table S1), where MlbF and MlbZ occur as over- and under-represented species, respectively, thus suggesting a control mechanism for the cellular compartmentalization of the latter protein. In addition, an over-representation of proteins generally related to cell detoxification and protective mechanisms against antibiotic toxicity was revealed. In particular, various ATPase components (NCBI accession number: ETK31443.1, ETK37841.1 and ETK33189.1), which are part of ABC-type multidrug transporter systems, as inferred by bioinformatics [42], were differentially represented in global and/or membrane proteome analysis at A substages (Additional file 1: Table S1 and Additional file 3: Table S3). Indeed, these components showed opposite accumulation profiles with the interesting exception of ETK33189.1 protein, which was over-represented in both global and membrane proteome analyses.

#### Stress response proteins

A differential representation of stress response proteins, such as TrxB and TrxA, catalase/peroxidase HPI, siroheme synthase, globin, UspA and phage shock protein A (PspA), was observed at A substages in global and/or membrane proteome analysis (Additional file 1: Table S1 and Additional file 3: Table S3). These proteins are generally induced as result of various environmental challenges [43–46]. In particular, an over-representation at A-90 h was observed for TrxB, siroheme synthase, globin, and UspA. PspA and HPI showed discordant and inverse accumulation levels in the whole/membrane bacterial extracts during NAI-107 production, being PspA modestly-increased/decreased and HPI modestly-decreased/increased, respectively. These results suggest a differential compartmentalization of these proteins. In the case of PspA, the compartment-dependent accumulation profile may be related with its dual role as regulatory element and membrane component, similarly to what already observed in *E. coli* [45].

### Comparative proteomic analysis between D substages characterized by different profile of NAI-107 yield

The D stage is characterized by a decrease in the amount of bacterial biomass compatible with cell growth arrest and cellular lysis occurrences (Fig. 1a). However, surviving cells are still metabolically active and use medium peptides as carbon and nitrogen source. This can be inferred from the progressive increment of the pH value of the medium (Fig. 1c), as consequence of amino acid utilization and ammonia production [24–26], and by the NAI-107 concentration that increases until D-140 h (Fig. 1a). In order to shed light on the molecular events associated with NAI-107 production maintenance and arrest, differential proteomic analysis was carried out on biomass samples collected at D-117, D-140 and D-162 h substages, using D-117 h as pivotal time-point. This analysis revealed 171 differentially represented spots in the D-117 *vs* D-140 h comparison, with 111 and 60 ones over- and under-represented at D-117 h, respectively (Additional file 2: Table S2); on the other hand, 265 differentially represented spots were observed in the D-117 *vs* D-162 h comparison, with 130 and 135 ones over- and under-represented at D-117 h, respectively. Hundred and fifty-one spots were in common between the two datasets showing concordant abundance profiles in terms of up- and down-regulation (Additional file 2: Table S2). Thus, 285 spots were then subjected to MS analysis for protein identification. Identified proteins were clustered according their function as reported above (Fig. 2c and d). Proteins occurring in spots (19 in total) presenting the concomitant presence of multiple components (Additional file 6: Table S5) were excluded from clustering. Functional analysis revealed that proteins with unknown function were the most represented group (i) of differentially regulated species, followed by that of species involved in carbon and protein metabolism (b and g, respectively). The two datasets showed a concordant profile and distribution into functional groups of over- and under-represented protein species (Fig. 3c and d). These results univocally revealed that proteins belonging to all functional groups were mostly over-represented at D-117 h (Fig. 3c and d; Fig. 4b); conversely, an opposite trend was observed for proteins with unknown function (under-represented at D-117 h). In particular, proteins involved in protein biosynthesis, folding and turn-over, stress response factors, transport through membranes (either involved in amino acid, peptide and sugars up-take), and enzymes active in nucleotide, energy and carbon metabolism (mostly in TCA cycle) were accumulated at D-117 h (Additional file 2: Table S2; Fig. 4b). Global accumulation pattern of these functional groups well paralleled the fact that mycelial cells stopped growth at D substages, with the occurrence of concomitant events of cell lysis which became more severe after D-140 h, thus determining the dramatic decrease of biomass content and NAI-107 production yield as observed thereafter (Fig. 1a). Interestingly, a number of proteins or specific metabolic/molecular processes already observed as over-represented at A-90 h confirmed their positive association with the progression of NAI-107 production in the D stages, resulting over-represented at D-117 h with respect to D-162 h or to both D-140 and D-162 h substages. Prototype examples are: i) cell wall biosynthesis/maturation (i.e., glutamine-fructose-6-phosphate transaminase and serine-type D-Ala-D-Ala carboxypeptidase); ii) lipid modification (i.e., CFA); iii) nitrogen metabolism (i.e., two GlnA paralogues and GlnK [36]); iv) oxidative (i.e. TrxB and superoxide dismutase) and osmotic stress response (i.e., OsmC [47, 48]); v) NAI-107 transport across membrane, (i.e. the above-mentioned MlbF and MlbZ ATPase); v) ABC-type multidrug transporter systems (i.e. NCBI accession number: ETK31443.1) (Additional file 2: Table S2).

### Construction and characterization of a *tetR* over-expressing *Microbispora* ATCC-PTA-5024 strain

A transcriptional regulatory protein of the TetR family (NCBI accession number: ETK34117.1) was observed as slightly over-represented at A-90 h, and progressively accumulating during the bacterial cultivation (Additional file 5: Figure S3). TetR family proteins generally act as transcriptional repressors; in the *Streptomyces* genus, they have been reported as being involved in many physiological processes [49], including antibiotic production [50, 51]. The relationship between the accumulation of the TetR-like transcriptional regulator and *Microbispora* ATCC-PTA-5024 physiological differentiation was analysed through the construction of a *tetR* over-expressing *Microbispora* ATCC-PTA-5024 strain by using the pIJ8600 plasmid containing a thiostrepton-inducible promoter upstream the corresponding cloning site [52]. In *Microbispora* ATCC-PTA-5024, over-expression of this gene promoted both morphological - i.e. formation of white aerial mycelium - and physiological differentiation – i.e. increased antibiotic production - (Additional file 5: Figure S4 and S5), thus confirming its important role in the progression of the *Microbispora* ATCC-PTA-5024 life cycle.

### Evaluation of NAI-107 impact on *Microbispora* growth physiology

The differential proteomic analyses performed in this study on WT strain revealed a pattern of accumulation of stress response proteins, including those involved in oxidative, osmotic and/or cell envelope-integrity stress response, in coincidence with NAI-107 production. In order to assess if this phenomenon could be directly imputable to NAI-107, a null mutant for NAI-107 production (RP0), constructed by *mlbA* disruption [6], was exposed to an externally-added NAI-107 amount during growth. In particular, the RP0 strain was incubated in KV6 medium, where the absence of NAI-107 was verified by LC-MS analysis. Then, NAI-107 was added to RP0 strain cultivations in coincidence with glucose consumption (at 72 h; Fig. 5a). Chosen NAI-107 concentration (20 mg/l) was similar to that observed for WT strain at the end of the A stage (Fig. 1a). Exposition of the bacterial cells to the lantibiotic for 24 h and thereafter resulted in mycelial cell lysis, as inferred by the decrease in the corresponding biomass content, in comparison with untreated cultivations (Fig. 5). The NAI-107 effects on *Microbispora* mycelium were evaluated by differential proteome analysis and confocal microscopy analysis coupled with differential fluorescent staining, as described in the following two sub-paragraphs.

#### Effects of the NAI-107 addition on the proteomic profile of Microbispora ATCC-PTA-5024 RP0 strain

RP0 strain biomass samples were collected at 96 h of incubation from either NAI-107 exposed and unexposed cultivations (Fig. 5) and then processed for differential proteomics. Additional file 7: Table S4 and Additional file 6: Table S5 report the identification of the differentially abundant protein species; description and representations of overall results of this analysis are in Additional file 4: Supplementary Results section, and in Figs. 2e and 3e, respectively. By cross-checking global proteomic data obtained from the experiments on *Microbispora* ATCC-PTA-5024 WT strain at A-66 h *vs* A-90 h (before and during NAI-107 production) and on NAI-107 exposed *vs* unexposed RP0 strain, worth mentioning was the observation of 19 proteins (out of the 33 in common) which have a concordant accumulation profile (Additional file 7: Table S4). Their quantitative trend well paralleled with the lantibiotic presence in the medium (Additional file 7: Table S4). Among them, proteins over-represented in presence of NAI-107 are two ATPase components of the ABC-type multidrug transporter systems (i.e. ETK31443.1 and ETK33189.1), CFA enzyme and PspA protein above mentioned.

#### Effects of the NAI-107 on cell membrane permeability

By using confocal microscopy coupled with differential fluorescent staining, a quantitative evaluation of the relative amount of damaged (red-labelled) on total (green-labelled) mycelium was provided in the WT strain at different time points (Additional file 5: Figure S6). In particular, a progressive increment of the relative amount of damaged mycelium was demonstrated during progression of NAI-107 accumulation (Fig. 1a), with a maximum observed at D-140 h (Additional file 5: Figure S6A) that preceded the further and pronounced decrease of biomass content due to cell lysis (Fig. 1a). This result confirmed a change in the membrane permeability that well paralleled with the accumulation of stress response factors therein, as revealed by proteomics. The fact that the alteration of the membrane structure was associated with NAI-107 presence was demonstrated by differential staining using confocal fluorescent microscopy on *Microbispora* ATCC-PTA-5024 RP0 strain after exposure to NAI-107 (Additional file 5: Figure S6). In fact, red intensity levels of NAI-107-treated RP0 cultures were similar to those of WT during NAI-107 yielding stages, while untreated cultivations did not show membrane permeability alterations at the same incubation time (Additional file 5: Figure S6).

## Discussion

In this study, proteomic investigations were carried out on *Microbispora* ATCC-PTA-5024, the producer of the promising lantibiotic NAI-107. In particular, a total of 303 gene products, participating into 241 molecular/metabolic functions, as inferred by a KEGG Orthology And Links Annotation (BlastKOALA) analysis [29] (Additional file 4: Supplementary Results; Additional file 8: Table S6), were identified and associated with NAI-107 production onset and maintenance during bacterial growth.

The NAI-107 production onset coincided with a pronounced decrease of glucose amount in the growth medium. At the molecular level, this phenomenon was associated with a down-representation of enzymes involved in glycolysis and oxidative phosphorylation, and of proteins involved in DNA replication, amino acid and protein biosynthesis; conversely, protein degradation factors as well as TCA cycle enzymes were instead over-represented during NAI-107 production at A-90 h (Fig. 5a). Interesting exceptions of the general trend of down-regulation of anabolic pathways at A-90 h were some over-represented enzymes involved in cell wall precursor biosynthesis. The glucose limitation signal may be responsible for a metabolic imbalance that results in the observed over-representation of oxidoreductive processes and oxidative stress response proteins (Additional file 1: Table S1 and Additional file 3: Table S3). In addition, the nutrient stress may be a triggering factor that, through the ppGpp activity (as suggested by ObgE GTPase and PNPase abundance profiles), can sequentially activate the *bld* cascade, as deduced by the accumulation profiles of the *Microbispora* BldK, BldG and BldD proteins. In agreement with this view, a stimulation of NAI-107 biosynthesis has been recently demonstrated in *Microbispora corallina* by the over-expression of *relA*, whose product is responsible of ppGpp biosynthesis [53]. Worth mentioning is the fact that the presence of BldK suggests the involvement of cell density signals too [38], which may integrate nutrient availability signals. Thus, in parallel with what already observed for the biosynthesis of the morphogenetic lanthionine containing peptide SapB in *S. coelicolor* [37, 54, 55], the *bld* regulatory cascade can promote the activation of pathway-specific *mlb* genes that control the production of NAI-107 in *Microbispora*. In line with these observations, products of the *mlb* cluster - i.e. ABC transporters MlbF and MlbZ, together with lantibiotic cyclase MlbC - accumulated in *Microbispora* ATCC-PTA-5024 during NAI-107 production at A-90 h. Interestingly, MlbF, MlbZ and MlbC were identified as associated with bacterial membranes, thus confirming the possibility that lantibiotic biosynthesis and self-immunity mechanisms occur in proximity of this bacterial district, as already reported in the case of nisin [28]. Indeed, proteomic analysis of the membrane extracts identified specific proteins, such as PspA, which shifted their quantitative representation moving from the cytosolic to the membrane district. These results confirmed also previous observations on cytosolic translational effectors in Gram-positive and Gram-negative bacteria, for which a “moon-ligthining” activity was claimed [56, 57].

Interestingly, the metabolism of nitrogen, phosphate and sulphur resulted important for NAI-107 production onset. Evidences of the importance of Pi for NAI-107 production have recently been reported [58]. In this study, the Pi content in the medium was observed as decreasing and reaching its minimum in coincidence of NAI-107 onset at A-90 h (Fig. 1b); then, it progressively increased during the D stage. This last evidence may be a consequence of two different and partly simultaneous events consistent with: i) mycelial cell lysis during the D stage - as inferred by several experimental evidences such as biomass content kinetics (Fig. 1a), fluorescent confocal microscopy observations (Additional file 5: Figure S6), the accumulation of osmotic and membrane-associated stress response factors (Additional file 1: Table S1, Additional file 2: Table S2, Additional file 3: Table S3) and overall diminished representation of primary metabolism enzymes during the transition from A to D stages (Additional file 5: Figure S1 and S2) - leading to the progressive release of cellular phosphate; ii) the scavenging action for phosphate from organic sources by Pho regulon gene products [35], whose members were observed as accumulating during NAI-107 production at A-90 h (Additional file 3: Table S3). This last occurrence well paralleled the Po consumption profile (Fig. 1b). On the other hand, the ammonia content profile suggests an equilibrium between production/assimilation of ammonia from medium peptides [24, 25], according with the activity of the GlnA enzyme [34] that was up-regulated at A-90 h (Additional file 1: Table S1). This equilibrium appeared as interrupted in coincidence with glucose exhaustion during D substages. Ammonia metabolism seems to play a crucial role also during NAI-107 production maintenance at D substages, due to the accumulation profile of two GlnA paralogues and of GlnK (Additional file 2: Table S2). Altogether, these evidences suggest that NAI-107 biosynthesis is mainly supported by the supply of general and essential nutrients, such as nitrogen, phosphate and sulphur. This is in good agreement with the fact that NAI-107 is a ribosomally synthesised lanthipeptide containing all the proteinogenic amino acids except phenylalanine, tyrosine, glutamine and histidine as can be inferred from the amino acid sequence of the precursor peptide encoded by the structural gene *mlbA*.

An interesting finding from comparing A and D substage proteomes was the increasing abundance of a TetR-like regulatory protein. The over-expression of this TetR-family regulator exerted a stimulatory effect on morphological and physiological differentiation in *Microbispora* ATCC-PTA-5024, as reported for the first time in this study. Although further studies are necessary to elucidate which regulatory circuits control the expression of this regulator and which genes are regulated by its activity, this result confirm the powerfulness of differential proteomic-based approaches to reveal regulatory factors playing crucial role in actinomycete biology.

Another common aspect that characterized A and D substage proteomes is the evidence of a positive correlation between NAI-107 production and the stimulation of cellular processes involved in NAI-107 transport, cell wall biosynthesis/maturation, lipid modification, stress response and multidrug resistance mechanisms. Their up-regulation during NAI-107 production suggests that, beside having *mlb* specific genes associated with self-immunity [6], *Microbispora* ATCC-PTA-5024 genome contains a number of additional genes whose increased expression possibly diminish/prevent the potential detrimental effects due to this lantibiotic. A similar phenomenon was described in *S. coelicolor* during the metabolic switching that leads to secondary metabolism activation [26], and also emerges from studies on other actinomycetes producing antibiotic metabolites [16, 17, 19, 21, 22]. This can be related to the general responses exerted by bacterial strains in circumstances where they are exposed to antibiotics [59, 60]. Worth mentioning is the fact that *Bacillus subtilis* in exponential growth phase, when exposed to NAI-107, showed the over-representation of proteins that coincide with or are strictly related to the molecular processes here described for *Microbispora* ATCC-PTA-5024 [61].

Accumulation of factors that may help maintaining the integrity and stability of the cell membrane, i.e. CFA that catalyzes the cyclopropane ring formation in phospholipid biosynthesis [62, 63], further confirms that NAI-107 can alter membrane district integrity by binding to bacterial lipid II [61]. Although no a real pore-forming mechanism can be claimed to justify the NAI-107 activity [61], the NAI-107 capacity of modifying bacterial membrane permeability was highlighted here by confocal microscopy observations coupled with differential fluorescent staining. Confirmatory results for the involvement of cellular factors involved in the response to membrane permeability alterations and of ABC-type multi drug transporter systems as additional self-resistance mechanisms were finally obtained from the RP0 strain proteomic analysis performed by comparing NAI-107-exposed cultivations with unexposed ones. This analysis allowed identifying proteins directly induced to face the presence of NAI-107 in the medium. Thus, analogously to other holistic studies realized on antibiotic producing or non-producing bacteria [59, 60] the data obtained from differential proteomic analyses in this study for *Microbispora* ATCC-PTA-5024 strains suggests that antibiotics generally do not affect only proteins directly involved in drug binding, trafficking or metabolism, but also components that have no apparent relation with the bioactive molecules itself. Altogether, these findings reveal the existence of a well-conserved antibiotic bacterial resistome [64, 65] that can in turn support the function of signalling molecules besides inhibitors in a dose dependent manner [66].

## Conclusion

**Fig. 6:**
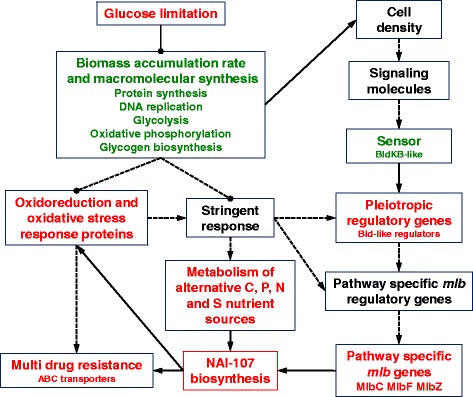
Signalling systems and regulatory cascades potentially controlling the physiological differentiation in *Microbispora* ATCC-PTA-5024. Red and green characters, highlighting molecular/metabolic processes in the corresponding boxes, stand for positive and negative association with NAI-107 production, respectively, according to the data reported in this study. Continuous and dashed lines stand for relationships inferred from this study and hypothetical ones based on scientific literature, respectively. Lines ending with arrow and black circle tips represent stimulatory and inhibitory relationships, respectively

This work reports the first proteomic study of the poorly investigated actinomycete *Microbispora* ATCC-PTA-5024 that produces the promising lantibiotic NAI-107. Patterns of protein accumulation/decrease during *Microbispora* ATCC-PTA-5024 growth provided a sharp and net contribution to the elucidation of the complex molecular, metabolic and regulatory pathways controlling physiological differentiation and eventually leading to NAI-107 production onset and maintenance. Integration of proteomic data with gene ontology results, fluorescence microscopy assays and analyses carried out on WT and genetically engineered strains allowed drawing a schematic representation of the regulatory pathways occurring in *Microbispora* ATCC-PTA-5024 (Fig. 6). The data reported in this study will contribute to the development of an efficient production process for NAI-107 through the application of rational genetic engineering approaches (i.e. the construction of *Microbispora* ATCC-PTA-5024 mutants knocked-in or -down in selected genes) and/or fermentation technology improvements (i.e. medium composition, pH control, concentration value optimization for Pi ammonia, sulphur and O_2_). It further supports the relevance and the powerfulness of proteomics in revealing novel players of antibiotic biosynthesis regulation in actinomycetes.

## Methods

### Strains, media and growth parameters

*Microbispora* ATCC-PTA-5024 strains were stored as frozen mycelium in GE82AB medium containing 20 % glycerol [58], at −80 °C. For proteomic analysis, strain cultivations were performed as previously described [58], with the second seed culture (6 % v/v) used for inoculating 100 ml of KV6 medium (12 g/l dextrose monohydrate, 12 g/l soy peptone, 12 g/l yeast extract, 2 g/l NaCl, pH 7.5) in a 500-mL baffled flask to get the appropriate biomass for subsequent analyses. *Microbispora* ATCC-PTA-5024 RP0 strain [6] was a gift from Prof. W. Wohlleben and Dr. E. Stegmann (University of Tubingen, Germany).

### Analysis of growth parameters

Biomass yield was determined by measuring the corresponding percentage packed biomass volume (PMV %), as obtained after centrifugation of a 6-ml culture sample at 4000 rcf, for 10 min, at 4 °C. Glucose concentration in the medium was evaluated by using a GM8 MicroStat analyzer (Analox) and the appropriate reagent, following manufacturer’s instructions. The pH value of the medium was measured using a 1-ml sample of spent medium and a Mettler-Toledo FiveGo pH-meter. The presence of NAI-107 congeners in the extracts was verified by LC-MS analysis and by microbiological assays as previously described [58]. Ammonia and Pi content in the medium was spectrophotometrically determined by Nessler reagent [67] and ammonium molybdate method [68], respectively. The Po content was measured as the increment of medium Pi content due to acid-hydrolysable phosphate released from organic sources after a treatment of spent medium aliquots with 5.25 N H_2_SO_4_ (1:12.5 v/v) in boiling water bath (30 min.) and subsequent neutralization with 5 N NaOH (1:10 v/v).

### 2D-DIGE analysis

Whole protein extracts were obtained from frozen biomass samples collected from three parallel flasks using the experimental procedure already described [17]. For extraction of membrane-associated proteins, bacterial mycelium was washed three times in Washing Solution (WS) and then disrupted by sonication in an ice bath [17]. Cell debris were removed by centrifugation at 12,000 rpm, for 15 min, at 4 °C; membranes in the supernatant were pelleted by ultracentrifugation at 150,000 g, for 30 min, at 4 °C. Membrane pellets were washed in WS containing 250 mM NaCl, and then repelleted by ultracentrifugation as above. An additional washing step was performed with WS alone. Membrane proteins were extracted by resuspending washed membranes in the appropriate IEF buffer [17] containing 2 % (w/v) ASB-14 (Sigma) and stored at -80 °C until use. All protein samples were labelled for 2D-DIGE analysis using the CyDyeTM DIGE minimal labelling kit (GE Healthcare, Sweden), which was used according to the manufacturer's instructions. Destreak Solution (DE, GE Healthcare) and DE plus 2 % (w/v) ASB-14 were used for isoelectrofocusing (IEF), which was performed on 3–10 non-linear pH range 18 cm-IPG strips (GE Healthcare) in an Ettan IPGphor III apparatus (GE Healthcare). After focusing, proteins were denatured, reduced and alkylated with iodoacetamide as previously described [17]. The 2D-gels were scanned with a DIGE imager (GE Healthcare) according to the manufacturer’s instructions. 2D-DIGE analysis was performed by using the Image Master 2D Platinum 7.0 DIGE software (GE Healthcare), according to the manufacturer’s instructions. Spots showing more than 1.3 fold change in spot volume (increased for over-representation or decreased for down- representation), with a statistically significance based on ANOVA test value (*P* ≤ 0.05), were considered as differentially represented and were further subjected to MS analysis for protein identification.

### Mass spectrometry analysis

Protein spots were carefully cut from the gels, triturated, *in gel*-reduced with dithiothreitol, S-alkylated with iodoacetamide, and subsequently *in-gel* digested with trypsin (Sigma). Resulting digests were desalted by μZip-TipC_18_ (Millipore) before MALDI-TOF-MS or nanoLC-ESI-LIT-MS/MS analysis [69]. In the first case, peptide mixtures were loaded on the MALDI target, using the dried droplet technique and α-cyano-4-hydroxycinnamic acid as matrix, and analyzed with a Voyager DE PRO mass spectrometer (Applied Biosystems, USA), operating in positive ion reflectron mode, with an acceleration voltage of 20 kV, a nitrogen laser (337 nm) and a laser repetition rate of 4 Hz. Mass spectra, measured over a mass range of 800–4,000 Da and by averaging 200-600 laser shots, were elaborated using the DataExplorer 5.1 software (Applied Biosystems) and manually inspected to obtain the corresponding peak lists. Internal mass calibration was carried out using peptides deriving from trypsin autoproteolysis. In the second case, digests were analyzed by nLC-ESI-LIT-MS/MS, using an LTQ XL mass spectrometer (ThermoFisher, USA) equipped with a Proxeon nanospray source connected to an Easy-nanoLC (Proxeon, Denmark). Peptides were resolved on an Easy C_18_ column (100 mm × 0.075 mm, 3 μm) (Proxeon, Denmark). Mobile phases were 0.1 % (v/v) formic acid (solvent A) and 0.1 % (v/v) formic acid in acetonitrile (solvent B), running at a total flow rate of 300 nl/min. A linear gradient was initiated 20 min after sample loading; solvent B ramped from 5 % to 35 % over 10 min, and from 35 % to 95 % over 2 min. Spectra were acquired in the range of *m/z* 400 − 2000. Peptides were analyzed under collision-induced dissociation data-dependent product ion scanning procedure, enabling dynamic exclusion (repeat count 1 and exclusion duration 60 s) over the three most abundant ions. Mass isolation window and collision energy were set to *m/z* 3 and 35 %, respectively.

### Protein identification

MASCOT search engine (version 2.2.06, Matrix Science, UK) was used to identify proteins within spots by searching the corresponding peptide peak lists against an NCBI non-redundant database (2014/01) also containing *Microbispora* ATCC-PTA-5024 ORF product database based on the corresponding genome sequence (GenBank accession AWEV00000000) [5]. Database searching of data from MALDI-TOF-peptide mass fingerprinting used a mass tolerance value of 40–80 ppm, trypsin as proteolytic enzyme, a missed cleavages maximum value of 2, and Cys carbamidomethylation and Met oxidation as fixed and variable modification, respectively. Other MASCOT parameters were kept as default. Database searching of data from nLC-ESI-LIT-MS/MS analysis used the same parameters reported above for proteolytic enzyme, missed cleavages maximum value, and fixed and variable modifications, respectively, but a mass tolerance value of 2.0 Da for precursor ion and 0.8 Da for MS/MS fragments. Definitive peptide assignment was always associated with manual spectra visualization/verification. Candidates from MALDI-TOF-MS analysis with a MASCOT score > 82, or from nLC-ESI-LIT-MS/MS analysis with at least 2 unique assigned peptides with an individual peptide expectation value < 0.05, in both cases corresponding to *P* < 0.05 (confidence level for protein identification > 95 %), were further evaluated by the comparison of their calculated mass and p*I* values with the experimental ones from 2-DE.

### Construction of the *Microbispora* ATCC-PTA-5024 proteome map database

Structure and design of web pages was realized in Hypertext Markup Language (HTML) and Cascading Style Sheets (CSS). Hypertext Preprocessor (PHP) was used to link the different gel views and build the connections to the database by following instruction described in PHP Manual [70]. To calculate spot position in the maps and make it interactive, PHP and JavaScript MooTools [71] were used. The database is structured in two tables “Spot information” and “Spot identification” for each experimental condition. Spot information includes protein biological/functional data and parameters from protein MS analysis. Spot identification includes spatial coordinates and information about spot quantification from the ImageMaster 2D Platinum software. A view combine the information of the two tables using the Experiment_ID and the Spot_ID. An additional table “Gel” includes the link to the pictures and is related to the other tables using the Experiment_ID.

### Differential fluorescence staining for confocal scanning laser microscope

Cell integrity was tested by differential staining using ethidium bromide (EB) [72], which was coupled with acridine orange (AO) for total cell staining [73]. In details, 100 μl of EB (5 μg/μl) and AO (5 μg/μl) mixture solution were added to 100 μl of mycelium suspension. After stirring by vortex for 30 s, the suspension was centrifuged at 13,000 rpm for 1 min, and the supernatant was discarded. Then, the pellet was suspended in 1 ml of 10 % (v/v) glycerol, 0.1 % (w/v) NaCl, stirred by vortex for 30 s, and finally centrifuged at 13000 rpm, for 3 min, for three times. Stained mycelium samples from replicate cultivations were visualized with FluoView-1000 laser scanning confocal microscopy (Olympus) using 60x objective lens magnification and the image analysis software Fluoview v.3.3. (Olympus). For quantitative analysis, at least three images (1024x1024 pixel) for each sample were randomly chosen, measured, analyzed and processed by the ImageJ software [74]. Resulting data were elaborated by Microsoft Excel™ software.

### Construction of the *tetR*-overexpressing *Microbispora* ATCC-PTA-5024 strain

*Microbispora* ATCC-PTA-5024 *tetR* gene was amplified by PCR (95 °C for 5 min; 95 °C for 1 min, 60 °C for 1 min, 72 °C for 1 min, for 40 cycles; 72 °C 10 min) using forward (*tetR*FOR 5’-CATATGGCTCGTGCGGGCCT-3’) and reverse (*tetR*REV 5’-GGATCCTCAGGCATTCCCCGC-3’) primers containing start and stop codons (in bold), and NdeI and BamHI recognition sites (underlined), respectively. The PCR product was digested with *Nde*I and *Bam*HI, and ligated into the pIJ8600 vector downstream the *tipA* promoter [52]. Ligation mixture was used to transform electrocompetent *E. coli* DH10B cells. The insert of the resulting recombinant plasmid (pIJ8600::*tetR*) was sequenced. After having transferred the pIJ8600::*tetR* in *E. coli* S17, this strain was used as donor in intergeneric conjugation with *Microbispora* ATCC-PTA-5024 WT strain as recipient. The recombinant colonies were selected by their Apr^R^ phenotype and the presence of pIJ8600::*tetR* was confirmed by PCR using primer pairs amplifying a pIJ8600 region containing the cloning site (ovFOR 5’-AGAGTTTGATCCTGGCTCA-3’ and ovREV 5’-AAGGAGGTGATCCAGCC-3’, as primers). Corrected site-specific integration of pIJ8600::*tetR* into *M.*. sp. ATCC-PTA-5024 chromosome was verified by Southern hybridization using *Sst*I digested pIJ8600 and pIJ::*tetR* plasmids as probes. A *Microbispora* ATCC-PTA-5024 strain carrying non-recombinant pIJ8600 was used as control strain. Plasmid pIJ8600 was a gift from Prof. Mervyn Bibb (John Innes Institute, Norwich, UK).

### Statistical analysis

Unless specifically stated, XLSTAT software was used for statistical analysis.

### Availability of supporting data

The data set supporting the results of this article are included in the main text, in the additional files and in the “*Microbispora* ATCC-PTA-5024 proteome web page” that can be found at the URL http://www.unipa.it/ampuglia/microbispora/.

## Additional files

Additional file 1: Table S1. Description, functional classification, abundance profile and mass spectrometry identification parameters of differentially represented *Microbispora* ATCC-PTA-5024 proteins identified from global proteome analysis at A substages. (XLSX 48 kb) Additional file 2: Table S2. Description, functional classification, abundance profile and mass spectrometry identification parameters of differentially represented *Microbispora* ATCC-PTA-5024 proteins identified from global proteome analysis at D substages. (XLS 107 kb) Additional file 3: Table S3. Description, functional classification, abundance profile and mass spectrometry identification parameters of differentially represented *Microbispora* ATCC-PTA-5024 proteins identified from membrane proteome analysis at A substages. (XLSX 37 kb) Additional file 4:
**Supplementary Results section.** (PDF 123 kb) Additional file 5:
**Figure S1-S6 with corresponding figure legends.** (PDF 511 kb) Additional file 6: Table S5. Description, abundance profile and mass spectrometry identification parameters of differentially represented spots containing multiple protein components. (XLS 45 kb) Additional file 7: Table S4. Description, functional classification, abundance profile and mass spectrometry identification parameters of differentially represented proteins due to NAI-107 exposure in *Microbispora* ATCC-PTA-5024 RP0 strain. (XLSX 32 kb) Additional file 8: Table S6. Numbers of KEGG orthology groups participating in molecular and metabolic processes as inferred from genome and proteome analyses, respectively. (XLS 24 kb)

## Abbreviations

2D-DIGE: 2D-Differential Gel Electrophoresis
A: Biomass accumulation
D: Biomass yield decline
IEF: Isoelectrofocusing
KO: KEGG orthology groups
MS: Mass spectrometry
Pi: Inorganic phosphate
PMV%: Percentage packed mycelium volume
Po: Hydrolysable phosphate from medium organic sources
Vol: Protein spot volume
