# Supplementary material for: Elucidating the molecular physiology of lantibiotic NAI-107 production in Microbispora ATCC-PTA-5024
Source: BMC Genomics. 2016 Jan 12;17:42. doi: 10.1186/s12864-016-2369-z (PMC4709908; doi:10.1186/s12864-016-2369-z)
Supplement: Additional file 4: — Supplementary Results section. (PDF 123 kb) [file 12864_2016_2369_MOESM4_ESM.pdf]

Elucidating the molecular physiology of lantibiotic NAI-107 production in *Microbispora* ATCC-PTA-5024

Giuseppe Gallo, Giovanni Renzone, Emilia Palazzotto, Paolo Monciardini, Simona Arena, Teresa Faddetta, Anna Giardina, Rosa Alduina, Tilmann Weber, Fabio Sangiorgi, Alessandro Russo, Giovanni Spinelli, Margherita Sosio, Andrea Scaloni and Anna Maria Puglia

Supplementary Results

Principal component analysis performed on protein abundance patterns A general decrease of acid proteins and an increment of basic ones was observed from A to D stages on the corresponding protein 2D-maps (Fig. S1). This observation is consistent with an overall diminished representation of primary metabolism enzymes during D substages. In fact, most *Microbispora* enzymes involved in primary metabolic processes have an acid p*I* value, as it has been shown by MS-based identification (Tab. S1-S3; Fig. S2). This observation is and in agreement with what can be inferred by other actinomycete proteome studies [S1-S3]. The protein abundance 2D-patterns gave also account for the bi-phasic trend of *Microbispora* ATCC-PTA-5024 WT biomass yield which paralleled glucose utilization firstly and glucose exhaustion in the medium then (Fig. 1).

Construction of an interactive web site of the proteomic 2D-maps *Microbispora* ATCC-PTA-5024 proteomic 2D-maps are provided in a digitalized database built as an interactive webpage available at http://www.unipa.it/ampuglia/microbispora/. This database correlates spots within 2D maps to protein entries within linked accession catalogues. For each spot, it is possible a visualization of the corresponding abundance value, experimental/theoretical p*I* and Mw values, MS identification procedure and results, and protein functional role according to the KEGG database [S4]. A total of 897 spots can be interrogated to obtain analytical, structural and biological information (Tab. S1-5). This database is easily expandable with novel data and represents a unique resource to store/manage *Microbispora* ATCC-PTA-5024 proteomic information.

Investigation of abundance profiles of protein species whose encoding genes are organized in putative operons An extensive investigation on proteomic results was performed taking into consideration the possible polycistronic organization of the genes encoding the differentially represented protein species. In particular, the putative polycistronic organization was inferred by a bioinformatic analysis carried-out on streptomycete homologous genes using the transcriptional organization description on BioCyc server [S5]. Thus, a detailed list of protein species whose

encoding genes are organised as putative operon and their corresponding fold change is reported below. In this list, protein proteolytic fragments, as deduced after the verification of their reduced Mw value with respect to intact counterpart (Tab. S1-4), were not included. Essentially, all the 108 protein species putatively co-transcribed revealed coherent profiles with the corresponding partners, with the exceptions of the following pairs: 50S ribosomal protein L4 (RPL4) and 30S ribosomal protein S10 (RPS10); 2-oxoglutarate ferredoxin oxidoreductase subunit alpha and beta; thioredoxin reductase (TrxB) and thioredoxin (TrxA); threonine aldolase and short-chain dehydrogenase/reductase SDR. For the different accumulation profiles of RPL4 and RPS10 proteins, post-transcriptional and translational regulatory events may be claimed, which are related to an autogenous regulation, where RPL4 acts as a transcriptional and translational negative regulator by binding the S10 operon transcript [S6]. For the different accumulation profile of TrxB and TrxA, whose abundances resulted unpaired also in the proteomic analyses of D-substage and RP0 mutant (Tab. S2 and S4), a different stability may be claimed, since TrxA is oxidized by many disulfide-bridge-containing proteins, especially under oxidative stress [S7]. For the other two protein pairs putatively co-transcribed, there are no evidences from scientific literature, which may explain the different profile. In this context, dedicated studies should be performed to address this point.

Protein species from Tab. S1 whose encoding genes are organized as putative operons.

Protein	Acronim	NCBI code	Fold change
chaperonin GroEL2	GroEL2	ETK30816.1	2.1
chaperonin GroEL2	GroEL2	ETK30816.1	2
chaperonin GroEL2	GroEL2	ETK30816.1	1.8
cold-shock DNA-binding domain-containing protein	CspA1	ETK30817.1	1.3
50S ribosomal protein L6	RPL6	ETK33161.1	-1.4
50S ribosomal protein L6	RPL6	ETK33161.1	-1.4
30S ribosomal protein S8	RPS8	ETK33162.1	-1.3
50S ribosomal protein L5	RPL5	ETK33164.1	-1.4

50S ribosomal protein L24	RPL24	ETK33165.1	-1.4
50S ribosomal protein L4	RPL4	ETK33175.1	1.5
30S ribosomal protein S10	RPS10	ETK33177.1	-1.4
elongation factor Tu	TufA	ETK33178.1	-1.5
elongation factor Tu	TufA	ETK33178.1	-1.6
elongation factor Tu	TufA	ETK33178.1	-1.6
elongation factor Tu	TufA	ETK33178.1	-1.7
translation elongation factor G	FusA	ETK33179.1	-2
translation elongation factor G	FusA	ETK33179.1	-2
translation elongation factor G	FusA	ETK33179.1	-2
ATP synthase F1 subunit epsilon	AtpC	ETK33856.1	-1.3
ATP synthase F1 subunit alpha	AtpA	ETK33859.1	-1.6
ATP synthase F1 subunit alpha	AtpA	ETK33859.1	-1.8
ATP synthase F1 subunit alpha	AtpA	ETK33859.1	-1.8
(protein-PII) uridylyltransferase	GlnD	ETK33939.1	1.3
nitrogen regulatory protein P-II	GlnK	ETK33940.1	4.3
nitrogen regulatory protein P-II	GlnK	ETK33940.1	3.7
translation elongation factor Ts	Tsf	ETK33965.1	-1.5
30S ribosomal protein S2	RPS2	ETK33966.1	-1.6
2-oxoglutarate ferredoxin oxidoreductase subunit beta	KorB	ETK34265.1	1.3
2-oxoglutarate ferredoxin oxidoreductase subunit alpha	KorA	ETK34266.1	-1.4
2-oxoglutarate ferredoxin oxidoreductase subunit alpha	KorA	ETK34266.1	-1.8
2-oxoglutarate ferredoxin oxidoreductase subunit alpha	KorA	ETK34266.1	-1.9
2-oxoglutarate ferredoxin oxidoreductase subunit beta	KorB	ETK34265.1	1.3
thioredoxin reductase	TrxB	ETK35037.1	1.5
Thioredoxin	TrxA	ETK35038.1	-1.3
Thioredoxin	TrxA	ETK35038.1	-1.6
DNA gyrase subunit B	GyrB	ETK35053.1	-1.3
DNA topoisomerase	GyrA	ETK35054.1	-1.4
proteasome subunit B	PrcB	ETK37956.1	1.6
proteasome subunit A	PrcA	ETK37957.1	1.4
transaldolase	Tal	ETK38137.1	-1.3
transketolase	Tkt1	ETK38138.1	-1.3
transketolase	Tkt1	ETK38138.1	-1.3
transketolase	Tkt1	ETK38138.1	-1.3

Protein species from Tab. S2 whose encoding genes are organized as putative operons.

Protein	Acronym	NCBI code	Fold change[a]	
elongation factor Tu	TufA	ETK33178.1	3.9	9.5
elongation factor Tu	TufA	ETK33178.1	2.9	5.6
elongation factor Tu	TufA	ETK33178.1	1.9	2.8
elongation factor Tu	TufA	ETK33178.1	2.4	3.8
translation elongation factor G	FusA	ETK33179.1	1.9	9
chaperonin GroEL	GroEL1	ETK33207.1	1.6	4.7
chaperonin GroEL	GroEL1	ETK33207.1	1.5	5.4
chaperonin GroEL1	GroEL1	ETK33207.1	1.4	1.7
chaperonin GroEL1	GroEL1	ETK33207.1	N. S.	5
co-chaperonin GroES	GroES	ETK33208.1	2.4	2.7
ATP synthase F1 subunit alpha	AtpA	ETK33859.1	2	2.6
ATP synthase F1 subunit alpha	AtpA	ETK33859.1	3.2	9.2
ATP synthase F1 subunit alpha	AtpA	ETK33859.1	1.6	2.7
ATP synthase F1 subunit alpha	AtpA	ETK33859.1	2.1	4.2
ATP synthase F1 subunit alpha	AtpA	ETK33859.1	3.5	7
ATP synthase F1 subunit alpha	AtpA	ETK33859.1	1.5	3.2
ATP synthase F1 subunit alpha	AtpA	ETK33859.1	N. S.	5.7
ATP synthase F1 subunit alpha	AtpA	ETK33859.1	N. S.	5
ATP synthase F1 subunit alpha	AtpA	ETK33859.1	N. S.	3.8
ATP synthase F1 subunit delta	AtpH	ETK33860.1	N. S.	1.6
translation elongation factor Ts	Tsf	ETK33965.1	N. S.	2.2
30S ribosomal protein S2	RPS2	ETK33966.1	1.8	4.7
NADH dehydrogenase (quinone)	NuoF	ETK34392.1	2	2.4
NADH-quinone oxidoreductase, chain G	NuoG	ETK34393.1	1.8	4.3
threonine aldolase	LtaE	ETK36049.1	N. S.	2
short-chain dehydrogenase/reductase SDR		ETK36050.1	N. S.	-1.8
transaldolase	Tal	ETK38137.1	2.2	3.5
transketolase	Tkt1	ETK38138.1	2.1	7.7
glyceraldehyde-3-phosphate dehydrogenase	GAPA	ETK38176.1	2.4	2.5
glyceraldehyde-3-phosphate dehydrogenase	GAPA	ETK38176.1	1.4	1.9
glyceraldehyde-3-phosphate dehydrogenase	GAPA	ETK38176.1	N. S.	1.9
phosphoglycerate kinase	PGK	ETK38177.1	1.8	3.9

[a] N. S.: Not significant (*e. g.* fold change below 1.3 threshold)

Protein species from Tab. S3 whose encoding genes are organized as putative operons.

Protein	Acronym	NCBI code	Fold change
elongation factor Tu	TufA	ETK33178.1	2.2
elongation factor Tu	TufA	ETK33178.1	2.3
elongation factor Tu	TufA	ETK33178.1	1.3
elongation factor Tu	TufA	ETK33178.1	2.4
elongation factor Tu	TufA	ETK33178.1	1.4
elongation factor Tu	TufA	ETK33178.1	1.7
translation elongation factor G	FusA	ETK33179.1	2.1
translation elongation factor G	FusA	ETK33179.1	1.9
translation elongation factor G	FusA	ETK33179.1	1.5
translation elongation factor G	FusA	ETK33179.1	2.9
translation elongation factor G	FusA	ETK33179.1	1.6
ABC transporter-like protein		ETK33304.1	-1.5
basic membrane lipoprotein		ETK33305.1	-2
basic membrane lipoprotein		ETK33305.1	-2.1
basic membrane lipoprotein		ETK33305.1	-1.9
ATP synthase subunit beta	AtpD	ETK33857.1	-1.7
ATP synthase F1 subunit alpha	AtpA	ETK33859.1	-1.5
ATP synthase F1 subunit delta	AtpH	ETK33860.1	-2
ATP synthase F1 subunit delta	AtpH	ETK33860.1	-2
UDP-N-acetylmuramate-alanine ligase	MurC	ETK36391.1	1.9
UDP-N-acetylmuramoylalanyl-D-glutamyl-2,6- diaminopimelate--D-alanyl-D-alanyl ligase	MurF	ETK36396.1	1.3

Protein species from Tab. S4 whose encoding genes are organized as putative operons.

Protein	Acronym	NCBI code	Fold change
ABC transporter-like protein		ETK33187.1	1.5
ABC transporter-like protein	NodI_2	ETK33189.1	1.7
ATP synthase subunit beta	AtpD	ETK33857.1	2.2
ATP synthase F1 subunit alpha	AtpA	ETK33859.1	2.2
ATP synthase F1 subunit alpha	AtpA	ETK33859.1	1.6
ATP synthase F1 subunit delta	AtpH	ETK33860.1	1.5
translation elongation factor Ts	Tsf	ETK33965.1	-1.6
30S ribosomal protein S2	RPS2	ETK33966.1	-1.5

Comparative proteomic analysis between A substages preceding and following lantibiotic production onset

Glucose catabolism enzymes Most of the differentially represented enzymes involved in carbon metabolism showed a decreased abundance at A-90 h substage (Fig. 4A). Among that, all glycolytic enzymes detected (phosphoglycerate mutase, phosphoglycerate kinase, glyceraldehyde-3-phosphate dehydrogenase, and pyruvate kinase) but fructose-bisphosphate aldolase, and enzymes using glycolysis products as substrates (phosphoenolpyruvate carboxylase and pyruvate dehydrogenase) showed a decreased abundance at A-90 h. Those species occurring as over-represented were generally associated with protein fragments deriving from proteolytic events, being their experimental mass by far lower than the theoretical one predicted for the intact component; this was the case of 2-oxoglutarate dehydrogenase E1 subunit, phosphoenolpyruvate carboxylase and pyruvate dehydrogenase (Tab. S1 and S3).

Protein metabolism According to the general down-representation of enzymes involved in amino acid metabolism during NAI-107 production at A-90 h, ribosomal proteins, protein chaperons (GroEL and heat shock proteins) and protein export-system components were generally down-represented in both global and membrane proteomic maps. On the other hand, many proteins associated with protein degradation and turn-over, such as ATPases, endopeptidase Clp, proteasome subunits A and B, resulted over-represented therein. When coupled with the reduced levels measured for RNA polymerase subunit RpoB, altogether these findings indicate a decrease of protein biosynthesis and secretion during NAI-107 production, which well paralleled with the corresponding promotion of protein degradation processes. This trend was also evident for some membrane-specific components, such as the ribosome-associated protein Y and Pup deaminase. The former component is a translational inhibitor associated with growth arrest [S8], while the latter one is an enzyme component activating the proteasome-dependent proteolysis [S9]. Beside protein-translocation system components, different proteins involved in aminoacyl-tRNA synthesis, protein

elongation and protein folding processes were also observed as associated with membranes (Tab. S3). Among them, only EF-Tu and EF-G proteins showed an opposite representation profile between the whole extract and membrane-specific proteome maps.

Nucleotide metabolism At A-90 h substage, proteomic analysis of whole bacterial extracts suggested a decreased representation of proteins involved in nucleotide metabolism, DNA replication and RNA synthesis such as the above mentioned polymerase subunit RpoB. Interestingly, some enzymes involved in nucleotide metabolism were observed as associated with membranes. Among that, most augmented ones were polypeptide fragments deriving from proteolysis of the corresponding intact species, for example, ribonucleoside-diphosphate reductase and DNA-directed RNA polymerase subunits beta and beta'. Interesting exceptions were uridylate kinase and a 5'-nucleotidase domain-containing protein, which are involved in the conversion of UDP into UMP and of nucleotides into the corresponding nucleosides, respectively. Altogether, these observations are consistent with a global metabolic context of decreased nucleotide synthesis.

Effects of the NAI-107 addition on the proteomic profile of *Microbispora* ATCC-PTA-5024 RP0 strain 2D-DIGE analysis revealed 144 differentially represented protein spots between NAI-107 exposed and unexposed samples. Among them, 47 and 97 spots were over- and down-represented as result of exposition to NAI-107, respectively. These 144 spots were treated and subjected to MS analysis for protein identification (Tab. S4); thirteen spots contained multiple proteins and were excluded from the subsequent analysis (Tab. S5). The remaining 131 ones were subjected to functional clustering according to what described above. Again, the most represented group of differentially represented components was that of proteins with unknown function, which was followed by the groups including enzymes involved in amino acid and carbohydrate metabolism (Fig. 2E). A quantitative evaluation of the differentially represented groups demonstrated that proteins with unknown function or involved in energy metabolism accumulated after NAI-107 exposure (Fig. 3E).

***Microbispora* ATCC-PTA-5024 genome analysis: overall molecular functions and metabolic capabilities** *Microbispora* ATCC-PTA-5024 genome contains 7918 coding sequences and it presents 21 potential clusters for the biosynthesis of secondary metabolites [S1, S10], including the *mlb* genes devoted to NAI-107 production. According to a gene ontology analysis performed by using KEGG Orthology And Links Annotation (BlastKOALA) [S4], 2695 coding sequences were assigned to 1555 KEGG orthology (KO) groups that participate into 1661 molecular/metabolic functions (Tab. S6). Functional analysis of the differentially represented proteins during bacterial growth identified 303 *Microbispora* ATCC-PTA-5024 gene products, which can be assigned to 198 KO groups participating into 241 molecular/metabolic functions (Tab. S6). They correspond to 14.5% of the cellular functions predicted for the *Microbispora* ATCC-PTA-5024 genome.

Supplementary Reference

S1) Tocchetti A, Bordoni R, Gallo G, Petiti L, Corti G, Alt S, Cruz JC, Salzano AM, Scaloni A, Puglia AM, De Bellis G, Peano C, Donadio S, Sosio M. **A Genomic, transcriptomic and proteomic look at the GE2270 producer *Planobispora rosea*, an uncommon actinomycete**. *PLoS One*, 2015, **10**(7):e0133705.

S2) Gallo G, Renzone G, Alduina R, Stegmann E, Weber T, Lantz AE, Thykaer J, Sangiorgi F, Scaloni A, Puglia AM. **Differential proteomic analysis reveals novel links between primary metabolism and antibiotic production in Amycolatopsis balhimycina**. *Proteomics*, 2010, **10**(7):1336-58.

S3) Hesketh AR, Chandra G, Shaw AD, Rowland JJ, Kell DB, Bibb MJ, Chater KF. **Primary and secondary metabolism, and post-translational protein modifications, as portrayed by proteomic analysis of *Streptomyces coelicolor***. *Mol Microbiol*, 2002, **46**(4):917-32.

S4) Kanehisa M, Goto S: **KEGG: kyoto encyclopedia of genes and genomes**. *Nucleic Acids Res* 2000, **28**(1):27-30.

S5) Caspi R, Altman T, Billington R, Dreher K, Foerster H, Fulcher CA, Holland TA, Keseler IM, Kothari A, Kubo A: **The MetaCyc database of metabolic pathways and enzymes and the BioCyc collection of Pathway/Genome Databases.** *Nucleic Acids Res* 2014, **42**(Database issue):D459-71.

S6) Zengel JM, Jerauld A, Walker A, Wahl MC, Lindahl L: **The extended loops of ribosomal proteins L4 and L22 are not required for ribosome assembly or L4-mediated autogenous control**. RNA, 2003, **9**(10):1188-97.

S7) Lu J, Holmgren A: **The thioredoxin antioxidant system.** *Free Radic Biol Med*, 2014, **66**:75-87.

S8) Agafonov DE, Kolb VA, Spirin AS: **Ribosome-associated protein that inhibits translation at the aminoacyl-tRNA binding stage**. *EMBO Rep* 2001, **2**(5):399-402.

S9) Yun HY, Tamura N, Tamura T: ***Rhodococcus* prokaryotic ubiquitin-like protein (Pup) is degraded by deaminase of pup (Dop)**. *Biosci Biotechnol Biochem* 2012, **76**(10):1959-1966.

S10) Sosio M, Gallo G, Pozzi R, Serina S, Monciardini P, Bera A, Stegmann E, Weber T: **Draft genome sequence of the *Microbispora* sp. strain ATCC-PTA-5024, producing the lantibiotic NAI-107**. *Genome Announc* 2014,**2**(1).
