# Supplementary material for: Elucidating the molecular physiology of lantibiotic NAI-107 production in Microbispora ATCC-PTA-5024
Source: BMC Genomics. 2016 Jan 12;17:42. doi: 10.1186/s12864-016-2369-z (PMC4709908; doi:10.1186/s12864-016-2369-z)
Supplement: Additional file 5: — Figure S1-S6 with corresponding figure legends. (PDF 511 kb) [file 12864_2016_2369_MOESM5_ESM.pdf]

Elucidating the molecular physiology of lantibiotic NAI-107 production in *Microbispora* ATCC-PTA-5024

Giuseppe Gallo, Giovanni Renzone, Emilia Palazzotto, Paolo Monciardini, Simona Arena, Teresa Faddetta, Anna Giardina, Rosa Alduina, Tilmann Weber, Fabio Sangiorgi, Alessandro Russo, Giovanni Spinelli, Margherita Sosio, Andrea Scaloni and Anna Maria Puglia

Supporting Figures 1-6

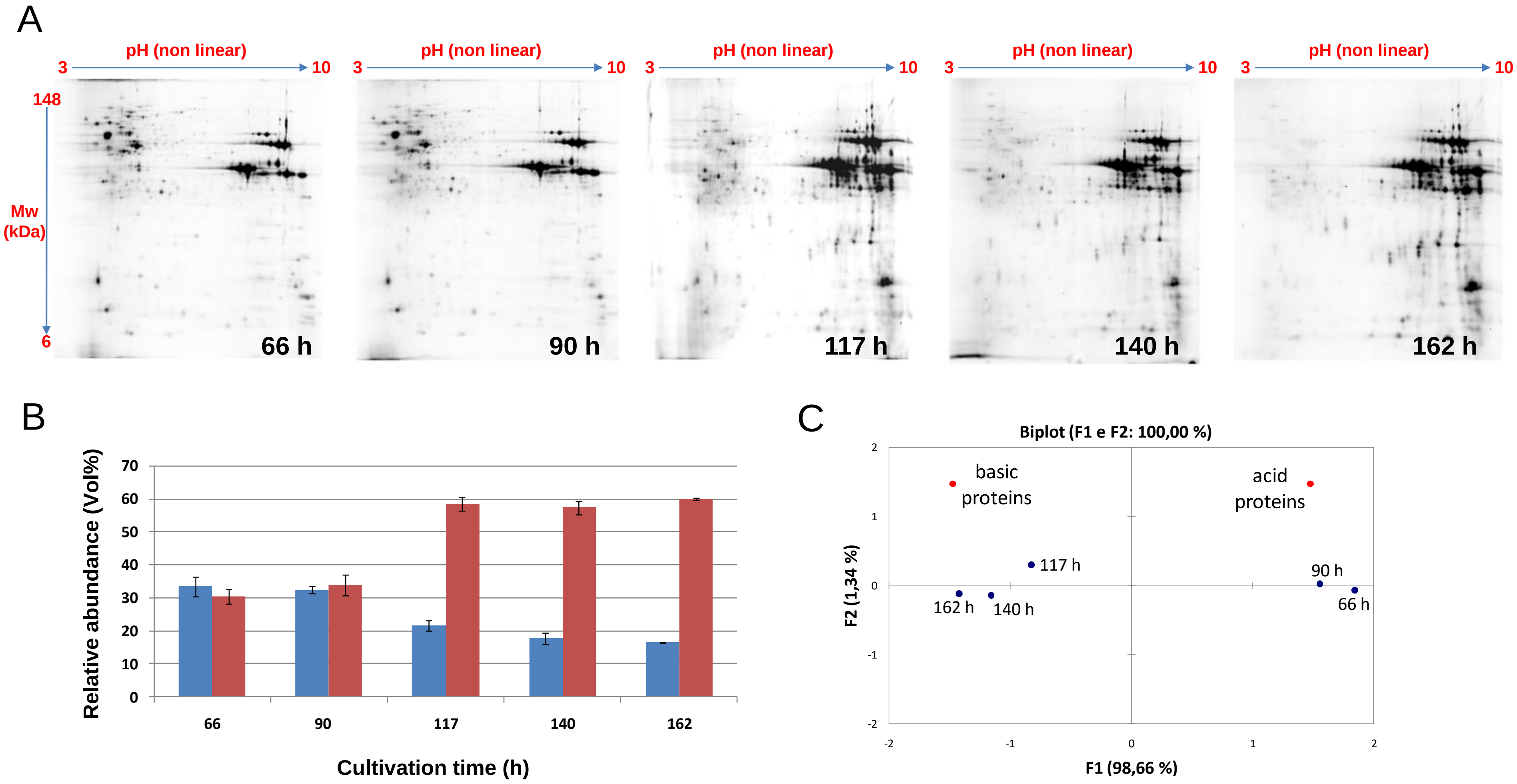

Figure S1. Proteomic analysis of whole protein extracts from *Microbispora* WT strain during cultivation. 2D-proteomic maps of *Microbispora* WT strain at the reported growth times (panel A). Relative abundance (measured as mean spot percentage volume - Vol%) ± S.D. of protein spots having acid (between 3-7, blue histograms) or basic (greater than 8, red histograms) isoelectric pH values (panel B). PCA analysis showing the correlation between the 2D-gel patterns at A and the D substages when reported in terms of overall abundance (Vol%) of acid and basic proteins.

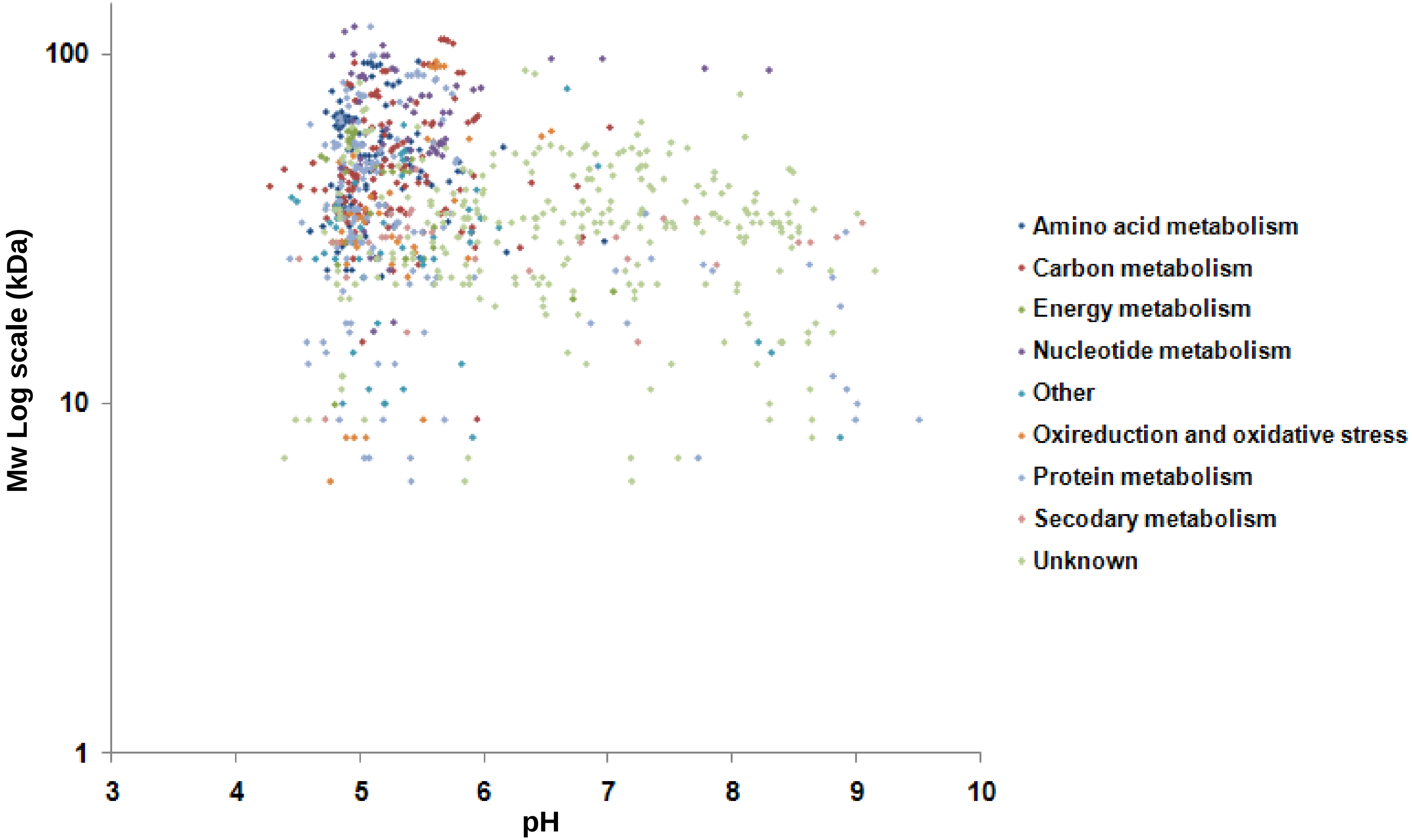

Figure S2. Experimental distribution of *Microbispora* protein species identified by MS procedures. The proteins are clustered according to the functional classification performed on the bases of KEGG ontology with the exception of some proteins grouped into "Secondary metabolism" class according to a bioinformatic analysis based on AntiSmash (Tab. S1-4).

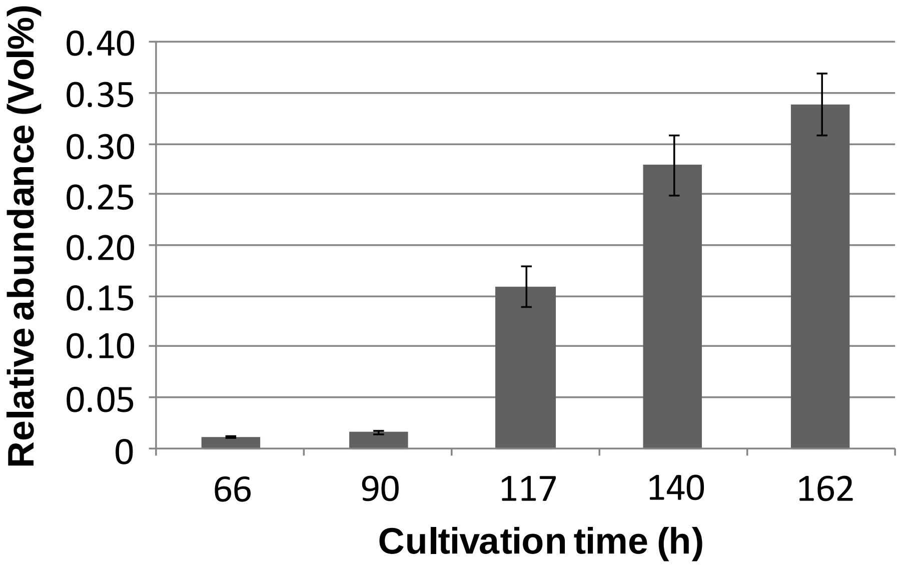

Figure S3. Accumulation of TetR-like protein during *Microbispora* WT strain cultivation. Quantitative values reported as mean spot percentage volume (Vol%) at different growth times calculated according to 2D-DIGE experiments. Vertical bars represent standard deviations.

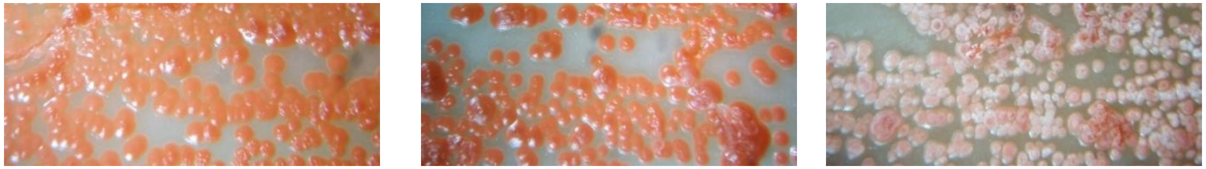

***Microbispora* sp.**
Wt strain

pIJ8600-containing
***Microbispora* sp. strain**

pIJ8600::*tetR*-containing
***Microbispora* sp. strain**

Figure S4. *Microbispora* strains grown on MS agar plate with thiostrepton sub-lethal amounts (50 ng/ml). Strains were incubated for 10 days. The formation of a white aerial mycelium is observable only in the *tetR* over-expressing strain, suggesting a role for this gene in morphological differentiations.

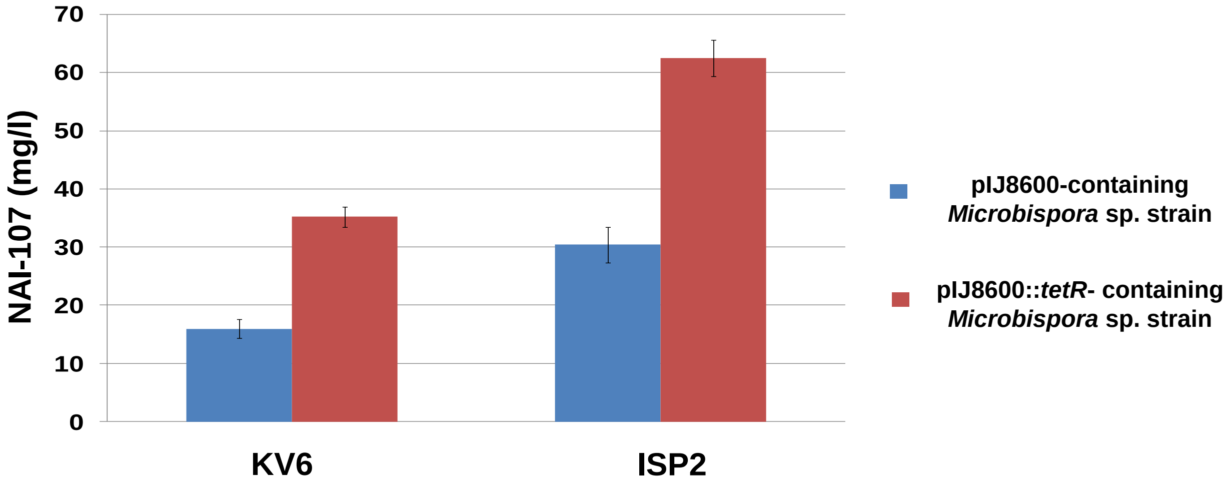

Figure S5. Antibiotic production of *Microbispora* strains in KV6 and ISP2 media. Methanolic extractions of NAI-107 were obtained from *Microbispora* sp. strains cultivated in 250 ml buffled flasks containing 20 ml of KV6 or ISP2 medium, at 30°C and 200 r.p.m. Cultures were grown for 4 days in presence of thiostrepton sub-lethal amounts (50 ng/ml). An increased antibiotic yield in *tetR*-overexpressing strain (red bars) was revealed with respect to control cultivation (blue bars); $P<0.05$ (ANOVA test). Histograms report mean values of bacterial inhibition-growth areas elicited by NAI-107 extracts for three independent cultivations. WT strain showed a behavior similar to that of the pI8600-containing *Microbispora* strain (data not shown). Vertical bars are representative of standard deviations.

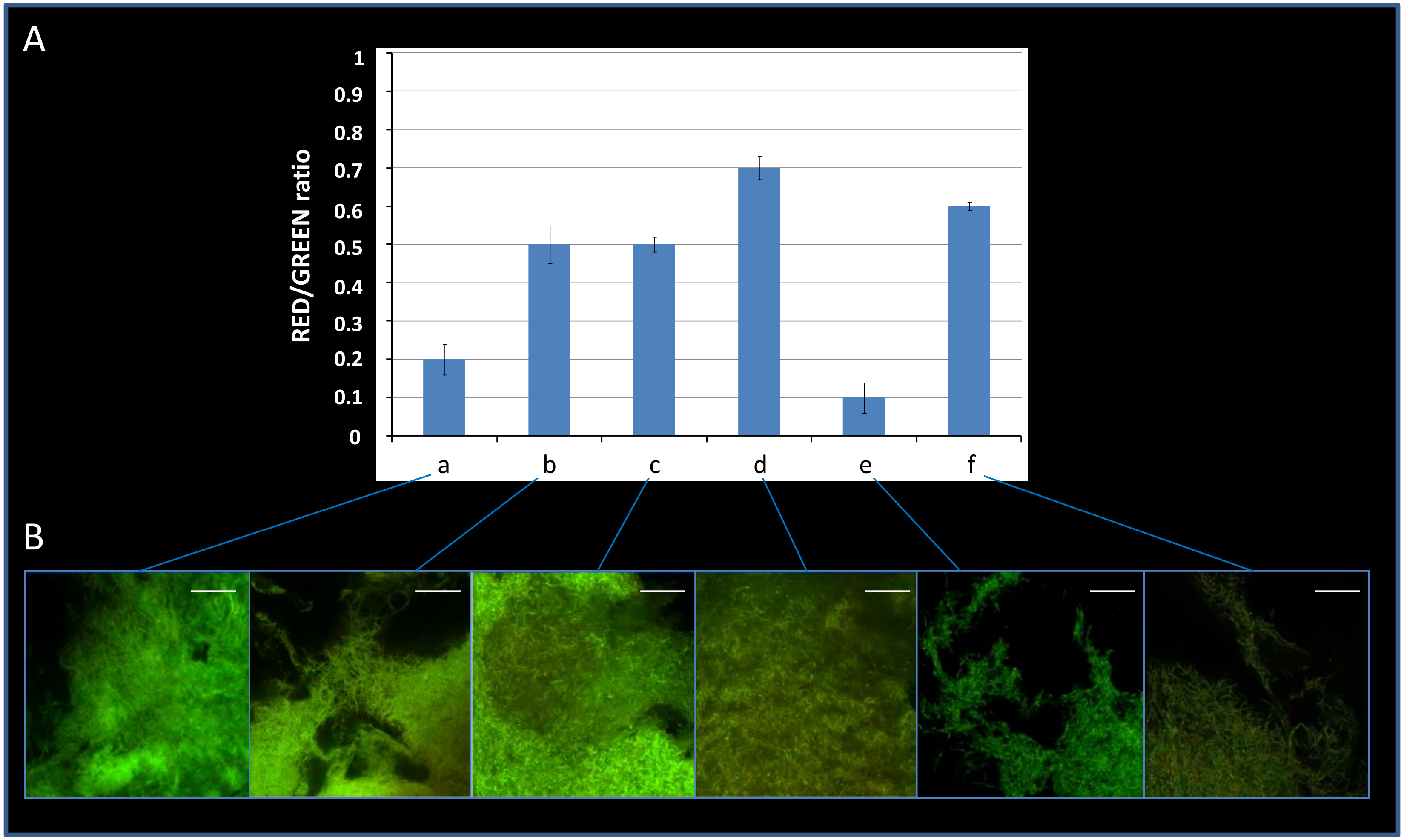

Figure S6. Confocal microscopy analysis of *Microbispora* WT and RP0 strains. Quantitative data (panel A) deriving from differential fluorescence staining micrographs (panel B) obtained with confocal microscopy. a: WT 66 h; b: WT 90 h; c: WT 117 h; d: WT 140 h; e: RP0; f: NAI-107-treated RP0. Red: ethidium bromide; green: acridine orange. White bars: 10 μm.
